# Distinguish *Dianthus* species or varieties based on chloroplast genomes

**DOI:** 10.1515/biol-2022-0772

**Published:** 2023-11-28

**Authors:** Dong Meng, Liu Yang, Zhao Yunlin, Yang Guiyan, Chen Shuwen, Xu Zhenggang

**Affiliations:** Hunan Provincial Key Lab of Dark Tea and Jin-hua, School of Materials and Chemical Engineering, Hunan City University, Yiyang 413000, Hunan, China; Hunan Research Center of Engineering Technology for Utilization of Environmental and Resources Plant, College of Life Science and Technology, Central South University of Forestry and Technology, Changsha 410004, Hunan, China; College of Forestry, Northwest A & F University, Yangling 712100, Shaanxi, China

**Keywords:** comparative analysis, simple sequence repeat, phylogenetic analysis, molecular markers

## Abstract

Most plants belonging to the widely distributed genus *Dianthus* are used for gardening. Interspecific hybridization of different *Dianthus* species leads to blurred genetic backgrounds. To obtain more genomic resources and understand the phylogenetic relationships among *Dianthus* species, the chloroplast genomes of 12 *Dianthus* species, including nine *Dianthus gratianopolitanus* varieties, were analyzed. The chloroplast genomes of these 12 species exhibited similar sizes (149,474–149,735 bp), with *Dianthus caryophyllus* having a chloroplast genome size of 149,604 bp marked by a significant contraction in inverted repeats. In the chloroplast genome of *Dianthus*, we identified 124–126 annotated genes, including 83–84 protein-coding genes. Notably, *D. caryophyllus* had 83 protein-coding genes but lacked *rpl2*. The repeat sequences of the chloroplast genome were consistent among species, and variations in the sequence were limited and not prominent. However, notable gene replacements were observed in the boundary region. Phylogenetic analysis of *Dianthus* indicated that *D. caryophyllus* and *D. gratianopolitanus* were most closely related, suggesting that the degree of variation within nine *Dianthus* varieties was no less than the variation observed between species. These differences provide a theoretical foundation for a more comprehensive understanding of the diversity within *Dianthus* species.

## 1 Introduction

*Dianthus* is a genus comprising approximately 300 species in the Caryophyllaceae family. Species within this genus are widely distributed across Europe and Asia, particularly in the Mediterranean region, as well as in America and Africa [1]. Several species, notably *Dianthus caryophyllus* L., *D. barbatus* Linn., *D. chinensi*s L., *D. plumarius* L., and *D. superbus* L., find extensive use in horticulture because of their beautiful flowers [1]. Moreover, many *Dianthus* species have been used in traditional Chinese medicine [2]. Two novel triterpenoid saponins with antimicrobial activity have been isolated from *D. erinaceus* Boiss. [3]. Furthermore, recent years have witnessed progress in research on *Dianthus* species, encompassing investigations into their morphological structure [4], molecular markers [5,6], environmental adaptation [7], and breeding techniques [1,8]. Because of the extensive utilization of *Dianthus* plants, the most commercially vital cultivars are hybrids that undergo vegetative propagation, and there has also been the development of transgenic varieties [8,9]. The use of advanced breeding technologies has expanded the diversity of *Dianthus* species, resulting in challenges when distinguishing these variations. This study underscores that morphological traits are the least effective means of identification, emphasizing the necessity for genetic markers in this regard [5].

Chloroplasts are vital organelles within green plant cells, closely involved in processes such as photosynthesis, carbon fixation, amino acid synthesis, and various other cellular functions [10]. In recent years, chloroplast DNA (CpDNA) has garnered increasing attention. According to the endosymbiotic theory, CpDNA is assumed to have originated from primordial photosynthetic prokaryotic cells associated with cyanobacteria [11]. CpDNA typically exhibits four regions: one large single-copy (LSC) region, one small single-copy (SSC) region, and a pair of inverted repeats (IRa and IRb) [12]. The absence of IRs in CpDNA is also widespread in many species, such as *Vicia sepium* [13]. CpDNA has single-parent genetic characteristics. Previous studies have shown that most gymnosperms inherit their chloroplasts paternally [14], whereas in angiosperms, chloroplast inheritance is primarily maternal [15]. Because of the lower replacement rates of CpDNA than that of nuclear genomes, CpDNA is frequently employed in the construction of evolutionary trees [16,17]. Extensive gene exchange occurs between the chloroplast genome, mitochondrial genome, and nuclear genomes, further diversifying the functions of chloroplasts [18–20]. The expression of genes encoded by CpDNA largely depends on a comprehensive set of factors of nuclear origin [21]. In recent years, CpDNA has been used to distinguish closely related species via comparative analysis [22–26].

*D. caryophyllus,* commonly known as carnation, is one the best-known species within the *Dianthus* genus [27]. The flowers of *D. caryophyllus* are renowned for their vibrant colors with strong aromas, including red, white, yellow, and green. The original natural flower color is a bright, pinkish-purple. They not only have ornamental value as cut flowers but also have medicinal properties. Our research team reported the characteristic CpDNA of *D. caryophyllus*, which is 147,604 bp long [28]. *D. gratianopolitanus* Vill., an endangered plant species, has a highly fragmented distribution range comprising numerous isolated populations [29]. To date, nine CpDNA sequences of *D. gratianopolitanus* varieties have been listed in the National Center for Biotechnology Information Database (https://www.ncbi.nlm.nih.gov/). However, there have been limited studies focusing on the genetic relationships and phylogenesis of *Dianthus* species. To explore the genetic characteristics of *Dianthus* species, a comprehensive comparison of CpDNA was conducted among different species and varieties of *Dianthus*. This study aims to test the hypothesis that differences in CpDNA characteristics among different species are greater than those among different varieties. The results of this study will not only enhance our understanding of the chloroplast genome characteristics of *Dianthus* but also provide valuable insights into their genetic diversity.

## 2 Materials and methods

### 2.1 Data collection and structure comparison

The CpDNA of *D. caryophyllus* was previously sequenced and published by our research team, under the accession number MG989277 [28]. A circular diagram was generated using OGDRAW 1.31 [30]. Similarly, 11 other *Dianthus* species were selected based on their nucleotide sequence similarity score using a BLAST search (https://blast.ncbi.nlm.nih.gov/). These species include *D. gratianopolitanus* (dg1602), *D. gratianopolitanus* (dg1869), *D. gratianopolitanus* (dg3051), *D. gratianopolitanus* (dg2134), *D. gratianopolitanus* (dg1856), *D. gratianopolitanus* (dg2769), *D. gratianopolitanus* (dg1255), *D. gratianopolitanus* (dg1370), *D. gratianopolitanus* (dg2868), *D. longicalyx* Miq., and *D. moravicus* Kovanda. The accession numbers for the CpDNA of all these plants are listed in Table 1. The GC content and length of the CpDNA as well as the LSC, SSC, and IR regions of these 12 species were calculated using BioEdit V7.0 [31]. The number of protein-coding genes was counted. Structural variations were detected using mVista in the shuffle-LAGAN model [32]. The sequences were visually checked for their identity using BLAST Ring Image Generator by aligning the 12 *Dianthus* species genomes with *D. caryophyllus* as a reference [33]. Simultaneous visual comparisons of the IR/LSC and IR/SSC border regions of the CpDNA from nine species were conducted using the drawing software Visio 2013, following the method described by Zhang et al. [34].

**Table 1:** Summary of chloroplast genomic for *D. caryophyllus* and other 11 Dianthus plants

Species (accession number)	LSC			SSC			IR			Total		Protein-coding genes
	Length (bp)	GC (%)	Length (%)	Length (bp)	GC (%)	Length (%)	Length (bp)	GC (%)	Length (%)	Length (bp)	GC (%)	
*D. caryophyllus* (MG989277)	84,774	34.13	57.43	17,100	29.77	11.59	22,865	42.78	15.49	149,604	36.30	83
*D. gratianopolitanus* dg1602 (LN877389)	82,954	33.98	55.40	17,123	29.75	11.44	24,829	42.44	16.58	149,735	36.30	84
*D. gratianopolitanus* dg1869 (LN877391)	82,938	33.97	55.39	17,168	29.66	11.47	24,809	42.44	16.57	149,724	36.29	84
*D. gratianopolitanus* dg3051 (LN877395)	82,959	33.98	55.43	17,121	29.77	11.44	24,795	42.46	16.57	149,670	36.31	84
*D. gratianopolitanus* dg2134 (LN877392)	82,906	33.99	55.39	17,131	29.69	11.45	24,814	42.44	16.58	149,665	36.30	84
*D. gratianopolitanus* dg1856 (LN877390)	82,945	33.99	55.42	17,117	29.77	11.44	24,800	42.45	16.57	149,662	36.31	84
*D. gratianopolitanus* dg2769 (LN877393)	82,871	34.01	55.40	17,095	29.83	11.43	24,815	42.45	16.59	149,596	36.33	84
*D. gratianopolitanus* dg1255 (LN877387)	82,831	34.01	55.37	17,138	29.81	11.46	24,808	42.45	16.58	149,585	36.33	84
*D. gratianopolitanus* dg1370 (LN877388)	82,805	34.02	55.38	17,120	29.70	11.45	24,793	42.45	16.58	149,511	36.32	84
*D. gratianopolitanus* dg2868 (LN877394)	82,768	34.03	55.37	17,118	29.71	11.45	24,794	42.45	16.59	149,474	36.33	84
*D. longicalyx* (MT001881)	82,804	34.04	55.37	17,127	29.78	11.45	24,804	42.44	16.59	149,539	36.34	84
*D. moravicus* (LN877396)	82,756	34.01	55.35	17,122	29.77	11.45	24,823	42.45	16.60	149,524	36.33	84

### 2.2 Analysis of simple sequence repeats (SSRs) and long repeat sequences

Repeat sequences are either identical or complementary, and occur repeatedly at different positions within the genome. Forward, reverse, complementary, and palindromic repeat sequences in CpDNA were predicted using REPuter software, with the minimal repeat size set to 20 bp [35]. Simultaneously, SSRs, also known as microsatellite repeat sequence were detected in the CpDNA of these plants, including hexanucleotide SSRs, using MISA (https://webblast.ipk-gatersleben.de/misa/) [36]. The minimum repeat units of genes were set as 10, 4, 4, 3, 3, and 3 for mononucleotide, dinucleotide, trinucleotide, tetranucleotide, pentanucleotide, and hexanucleotide unit sizes, respectively (Table 2).

**Table 2:** List of genes encoded by *D. caryophyllus* chloroplast genome

Group of genes	Gene names
Photosystem I	*psaA*, *psaB*, *psaC*, *psaI*, *psaJ*
Photosystem II	*psbA*, *psbB*, *psbC*, *psbD*, *psbE*, *psbF*, *psbH*, *psbI*, *psbJ*, *psbK*, *psbL*, *psbM*, *psbN*, *psbT*, *psbZ*
Cytochrome b/f complex	*petA*, *petB*, *petD*, *petG*, *petL*, *petN*
ATP synthase	*atpA*, *atpB*, *atpE*, *atpF* ^(a)^, *atpH*, *atpI*
NADH dehydrogenase	*ndhA* ^(a)^, *ndhB* (×2) ^(a)^, *ndhC*, *ndhD*, *ndhE*, *ndhF*, *ndhG*, *ndhH*, *ndhI*, *ndhJ, ndhK*
RubisCO large subunit	*rbcL*
RNA polymerase	*rpoA*, *rpoB*, *rpoC1* ^(a)^, *rpoC2*
Ribosomal proteins (SSU)	*rps11*, *rps12* (×2), *rps14*, *rps15*, *rps16*, *rps18*, *rps19*, *rps2*, *rps3*, *rps4*, *rps7* (×2), *rps8*
Ribosomal proteins (LSU)	*rpl14*, *rpl16*, *rpl2* ^(a)^, *rpl20*, *rpl22*, *rpl32*, *rpl33*, *rpl36*
Proteins of unknown function	*ycf1* (×2) ^(a)^, *ycf2* (×2), *ycf3* ^(b)^, *ycf4*
Transfer RNAs	*trnA-UGC* (×2), *trnC-GCA*, *trnD-GUC, trnE-UUC, trnF-GAA, trnfM-CAU, trnG-UCC, trnH-GUG, trnI-GAU* (×2), *trnK-UUU, trnL-CAA* (×2), *trnL-UAA,trnL-UAG, trnM-CAU, trnN-GUU* (×2), *trnP-UGG, trnQ-UUG, trnR-ACG* (×2), *trnR-UCU, trnS-GCU, trnS-GGA, trnS-UGA, trnT-GGU*, *trnT-UGU*, *trnV-GAC* (×2), *trnV-UAC, trnY-GUA*, *trnY-GUA*
Ribosomal RNAs	*rrn16* (×2), *rrn23* (×2), *rrn4.5* (×2), *rrn5* (×2)
Other genes	*infA*, *matK*, *clpP* ^(b)^, *accD*, *ccsA*, *cemA*

Note: (×2) means two gene copies; (a) means gene containing a single intron; (b) means gene containing two introns.

### 2.3 Analysis of codon usage bias

Codon usage bias reflects the balance between mutation bias and natural selection. In this study, we described the codon usage bias of CpDNA in all 12 selected plants using the relatively synonymous codon usage (RSCU) values, which were determined using DAMBE software [37]. An RSCU value of 1.00 indicated that the codons had no usage bias, whereas a value greater than 1.00 indicated a higher than expected usage frequency of the codon. To illustrate the distribution of codon usage preferences among these plants more clearly and intuitively, a heatmap based on the RSCU values using HemI software (version 1.0) was constructed [38].

### 2.4 Analysis of molecular evolution

Ka/Ks represents the ratio between the number of nonsynonymous substitutions per non-synonymous site (Ka) and the number of synonymous substitutions per synonymous site (Ks) in two protein-coding genes. It is used to determine whether there is selective pressure on the protein-coding gene [17]. If Ka/Ks > 1, the gene is considered to have undergone positive selection. If Ka/Ks = 1, the gene is considered to have evolved neutrally. If Ka/Ks < 1, the gene is considered to have undergone purify selection. Ka/Ks was calculated using *D. caryophyllus* as the reference, and the DnaSP v5 software was employed [39].

### 2.5 Phylogenetic analysis

The MEGA 7 software was employed to construct the phylogenetic tree using the neighbor-joining method, which is a simplified version of the minimal evolution method, for the aforementioned 12 CpDNAs [40]. The Kimura two-parameter model was selected using the corrected Akaike information criterion, and the bootstrap number was set to 100,000. The minimum value of the sum of all branches (S) was used as an estimate of the correct phylogenetic tree.

## 3 Results and discussion

### 3.1 CpDNA characteristics of *Dianthus* species

A comparison of the nine different varieties of *D. gratianopolitanus* revealed that although there were similarities in genome length, GC content, and the number of CpDNA genes, some differences persisted (Table 1). The CpDNA lengths of these nine varieties ranged from 149,474 bp (*D. gratianopolitanus* dg2868) to 149,735 bp (*D. gratianopolitanus* dg1602), with minimal variation. Moreover, the GC content of the nine CpDNAs ranged from 36.29 to 36.33%, with the IR regions showing relatively high GC content. *D. gratianopolitanus* (dg1869) had 124 genes, whereas the other eight varieties had 125 genes. All *D. gratianopolitanus* varieties contained 84 protein-encoding genes. It is worth mentioning that although the IR region of *D. caryophyllus* was the shortest in all 12 CpDNA, it exhibited the highest GC content. *D. caryophyllus* had the lowest number of protein-coding genes, totaling 83, and lacked the *rpl2* gene. Conversely, the largest number of genes was observed for *D. longicalyx,* with 126 genes.

### 3.2 Comparative genomic analysis of the *Dianthus* complete chloroplast genomes

**Figure 1:**
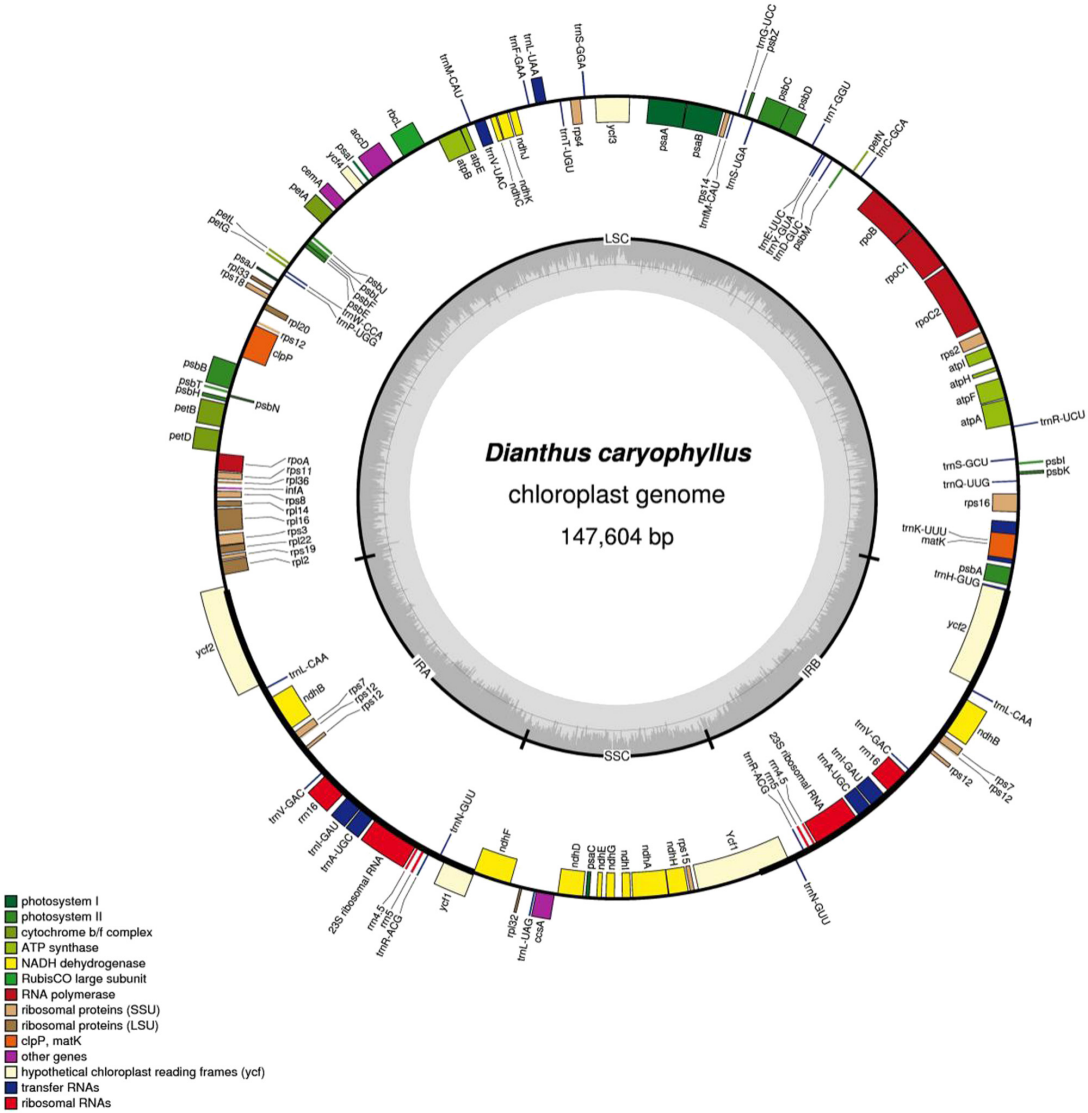
Circular map of the chloroplast genome of the *D. caryophyllus.* Genes drawn within the circle are transcribed clockwise, while genes drawn outside are transcribed counterclockwise. Genes belonging to different functional groups are color coded. Dark bold lines show inverted repeats (IRa, and IRb). The dashed area in the inner circle indicates GC content in the chloroplast genome. The map is drawn by OGDRAW.

**Figure 2:**
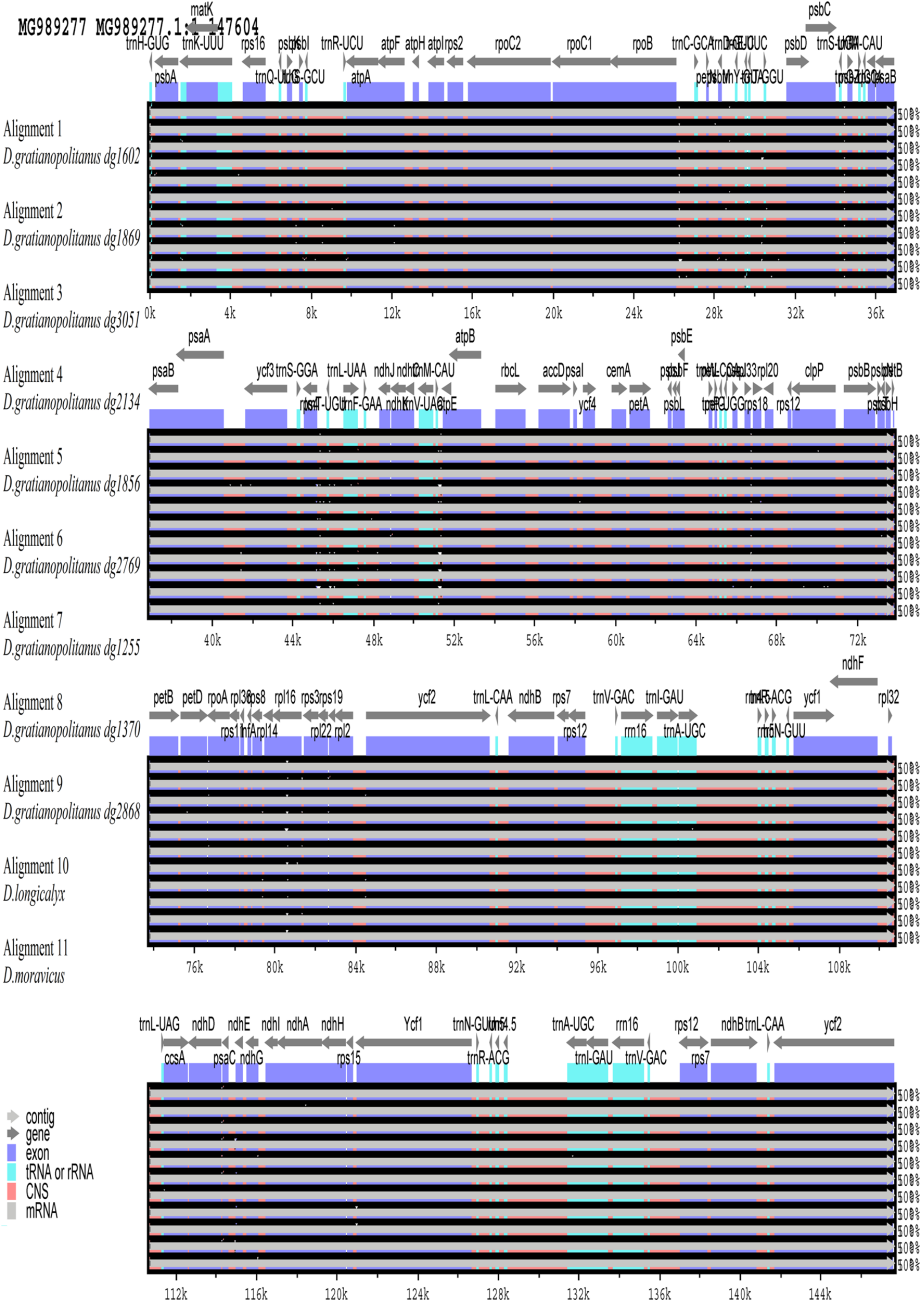
Visual alignments of chloroplast genome sequences using the *D. caryophyllus* as the reference genomes. Arrows indicate the annotated genes and their transcriptional direction, and genome regions are color coded as exon, tRNA or rRNA, CNS (conserved non-coding sequences), and mRNA.

Via multiple sequence alignment analysis of the nine *D. gratianopolitanus* varieties (Figure 1), we observed significant deviations primarily in the conserved non-coding sequences (CNS), tRNA, and rRNA. Notably, variant sites in the protein-coding sequences were primarily concentrated in the *atpF* gene in *D. gratianopolitanus* (dg1255) and *D. gratianopolitanus* (dg1370), the *ycf3* and *petD* genes in *D. gratianopolitanus* (dg3051), and the *clpP* gene in *D. gratianopolitanus* (dg1602) and *D. gratianopolitanus* (dg2868). Compared to the protein-coding sequence of *D. caryophyllus*, which exhibited notable distinctions in *clpP*, *atpF*, *ycf3*, the most significant divergence was observed in *rpl16*. Analyzed accessions of other 11 *Dianthus* species displayed conspicuous deletions in these gene. Overall, the dissimilarities between *D. caryophyllus* and *D. gratianopolitanus* (dg1869) were relatively minor, and their gene sequences are high degree of similarity. A comparative circular graph visually highlights the presence of genes, deletions, and sequence variations. The results indicate a high degree of sequence conservation, among the genomes (Figure 2).

**Figure 3:**
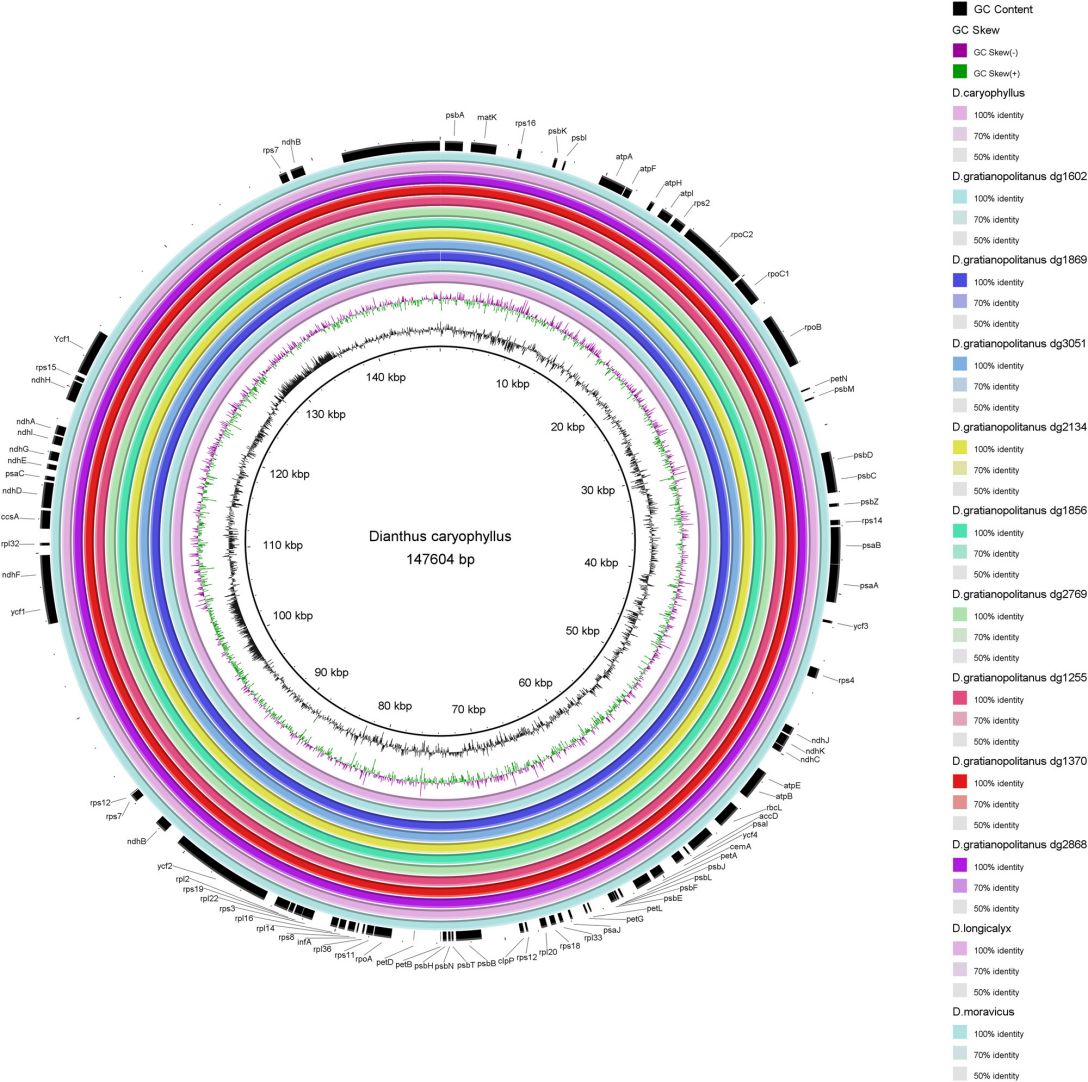
Comparative circle graph of similarity of the chloroplast genome sequences. The central ring represents the *D. caryophyllus* as the reference genome. There are 11 close related species plants from the inside to the outside. The outermost circle is the protein-coding gene of the *D. caryophyllus*.

Comparing the genes at the boundaries of the LSC, SSC, and IR regions (Figure 3), it becomes evident that the most significant difference among the nine *D. gratianopolitanus* varieties lies in the *psbA* (1,062 bp) within the LSC region of *D. gratianopolitanus* (dg1869). This same position was observed for *trnH* in eight other varieties. Although there were minor variations in the CpDNA of the nine *D. gratianopolitanus* species, overall, the CpDNA exhibited a high degree of conservation.

**Figure 4:**
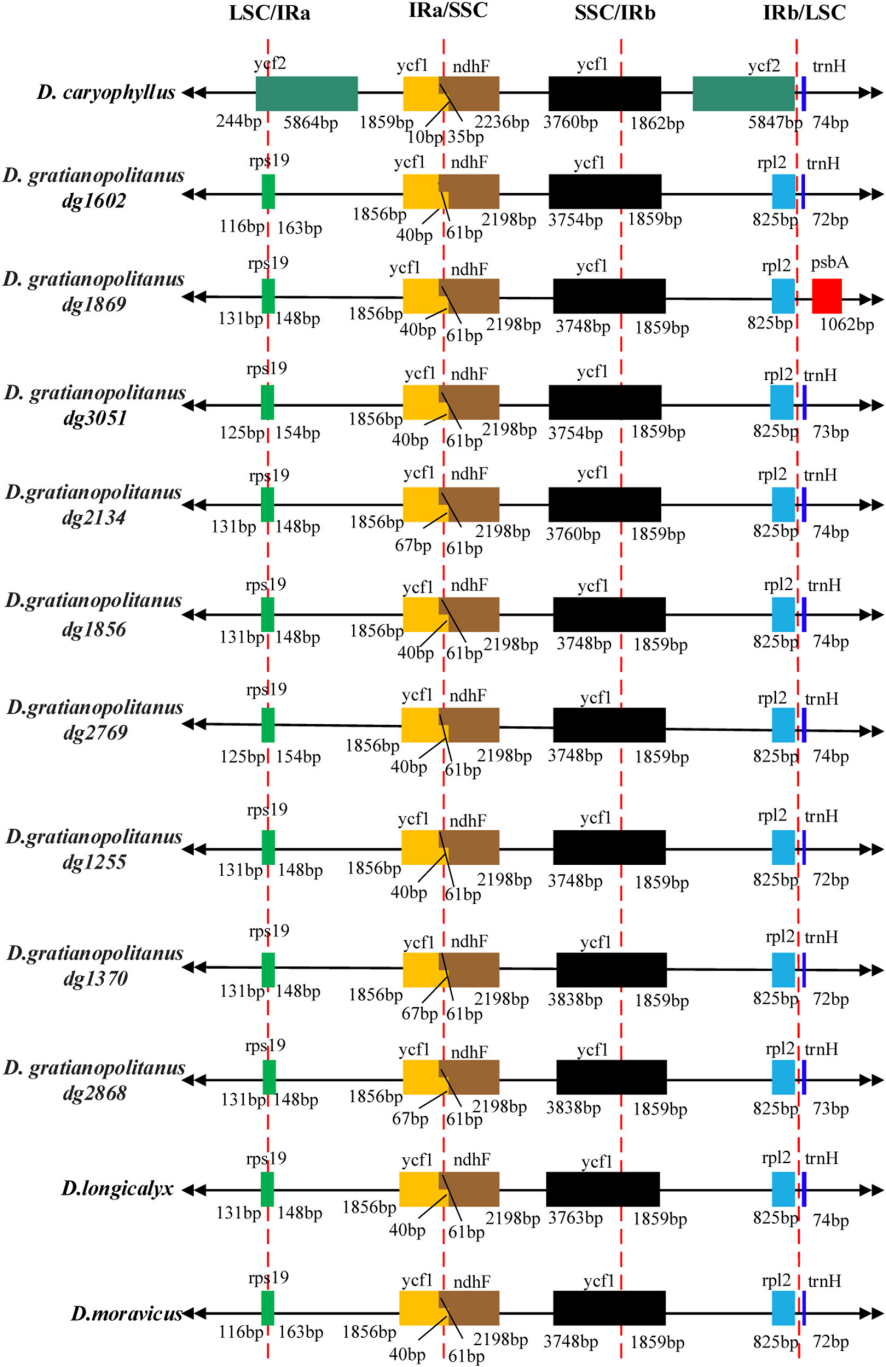
Comparison of the junctions of the four different constituent sequences in the chloroplast genome.

Figure 3 also highlights substantial differences between *D. caryophyllus* and the other 11 species in terms of genes at the boundaries of different regions. Notably, the LSC/IRa junction features *ycf2*, whereas *rps19* occupied this position in the other 11 species and had a similar segmentation length. In the IRb/LSC junctions, a duplicated *ycf2* gene replaces the *rpl2* gene, whereas the *rpl2* gene appears at the same connections as in other species. The *ycf1* gene in the IRb region is slightly longer in *D. caryophyllus*, differing by just 3 bp among the other 11 analyzed accessions chloroplast genomes (Figure 4).

### 3.3 Repeat sequence features

**Figure 5:**
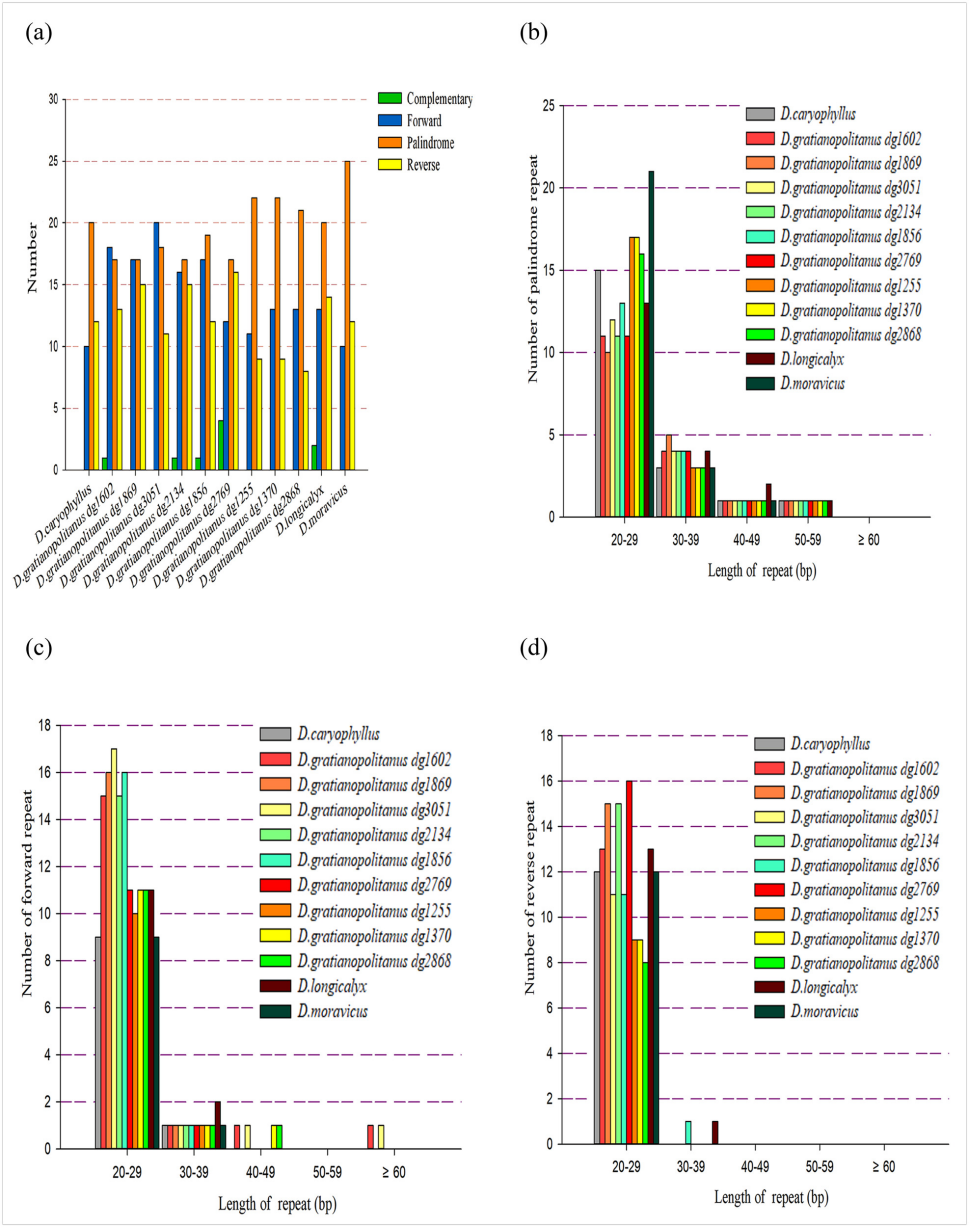
Long repeat sequence analysis in the chloroplast genome: (a) number of four different directions, (b) number of the palindrome directions repeat length, (c) number of the forward directions repeat length, and (d) number of the reverse directions repeat length.

Repeat sequence analysis was conducted on the CpDNA of the nine *D. gratianopolitanus* varieties, resulting in the identification of 49, 49, 49, 49, 49, 49, 42, 44, and 42 repeat sequences, respectively. These numbers were similar to those found in *D. longicalyx* (49) and *D. moravicus* (47) but were generally higher than those of the reference species, *D. caryophyllus*, which had only 42 repeat sequences (Figure 5a). Not all species contain all four types of repeat sequences. For instance, *D. caryophyllus* had only 10 forward, 20 palindromic, and 12 reverse repeat sequences. Only five species contained a very limited number of complementary repeat sequences, with *D. gratianopolitanus* (dg2769) having the most, but still only up to four. In contrast, the average numbers of forward, reverse, and palindromic repeats detected in the 12 CpDNAs were as high as 14, 12, and 20, respectively. As depicted in Figure 5b–d, palindromic repeat sequences were the most numerous, followed by forward and reverse repeats. Additionally, the sequence length of palindromic, forward, and reverse repeats was primarily concentrated in the 20–29 bp, followed by the 30–39 bp range. Repeat sequences longer than 59 bp, which are exclusively forward repeated sequences, were detected in *D. gratianopolitanus* (dg1602) and *D. gratianopolitanus* (dg3051).

**Figure 6:**
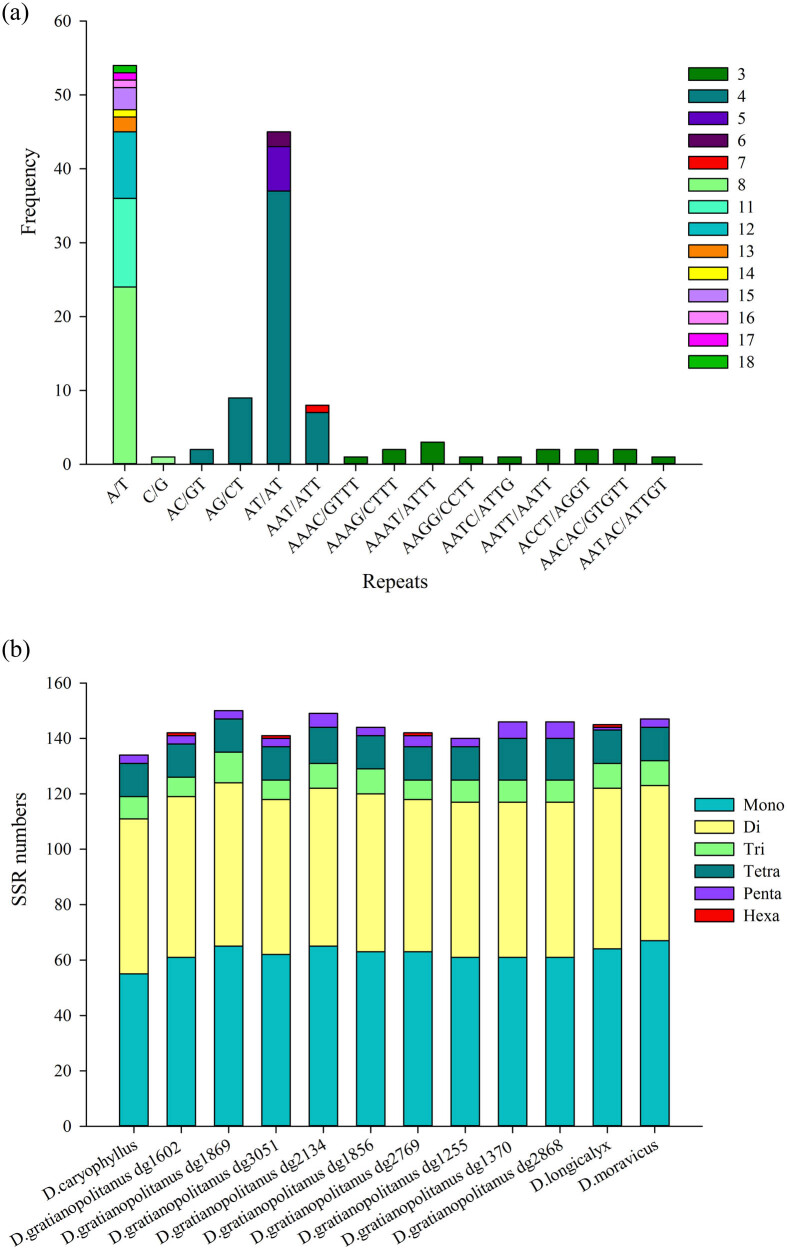
Comparison of the repeat sequence features of the chloroplast genome. (a) SSR types and corresponding cumulative frequency in *D. caryophyllus*. (b) Differences of the chloroplast genomic SSR between *D. caryophyllus* and other 11 close related species.

SSR analysis identified a total of 134 SSRs with 13 combined types in *D. caryophyllus* CpDNA (Figure 6a). Among these 134 SSRs, 55 mononucleotide, 56 dinucleotide, 8 trinucleotide, 12 tetranucleotide, and 3 pentanucleotide repeats but no hexanucleotide repeats were detected. Furthermore, the frequencies of short polyadenine or polythymine repeats were significantly higher than those of tandem cytosine (C) or guanine (G) repeats. The most frequent composed dinucleotide repeat was AT/TA. In addition, the CpDNA of the nine *D. gratianopolitanus* varieties contained 142, 150, 141, 149, 144, 142, 140, 146, and 146 SSRs, which were similar in number to those in *D. longicalyx* (145) and *D. moravicus* (147). Among the six types of SSR sequences in the 12 species, mononucleotide and dinucleotide repeats were the most common, with the largest numbers ranging from 55 to 67 (Figure 6b). The numbers of trinucleotide and tetranucleotide repeats were similar, whereas pentanucleotide repeats were less frequent, with each species averaging only approximately four. Hexanucleotide repeats were detected in the CpDNA of only four species, with each species having only one, except for *D. caryophyllus*, which did not contain any hexanucleotide repeats.

### 3.4 Codon usage

**Figure 7:**
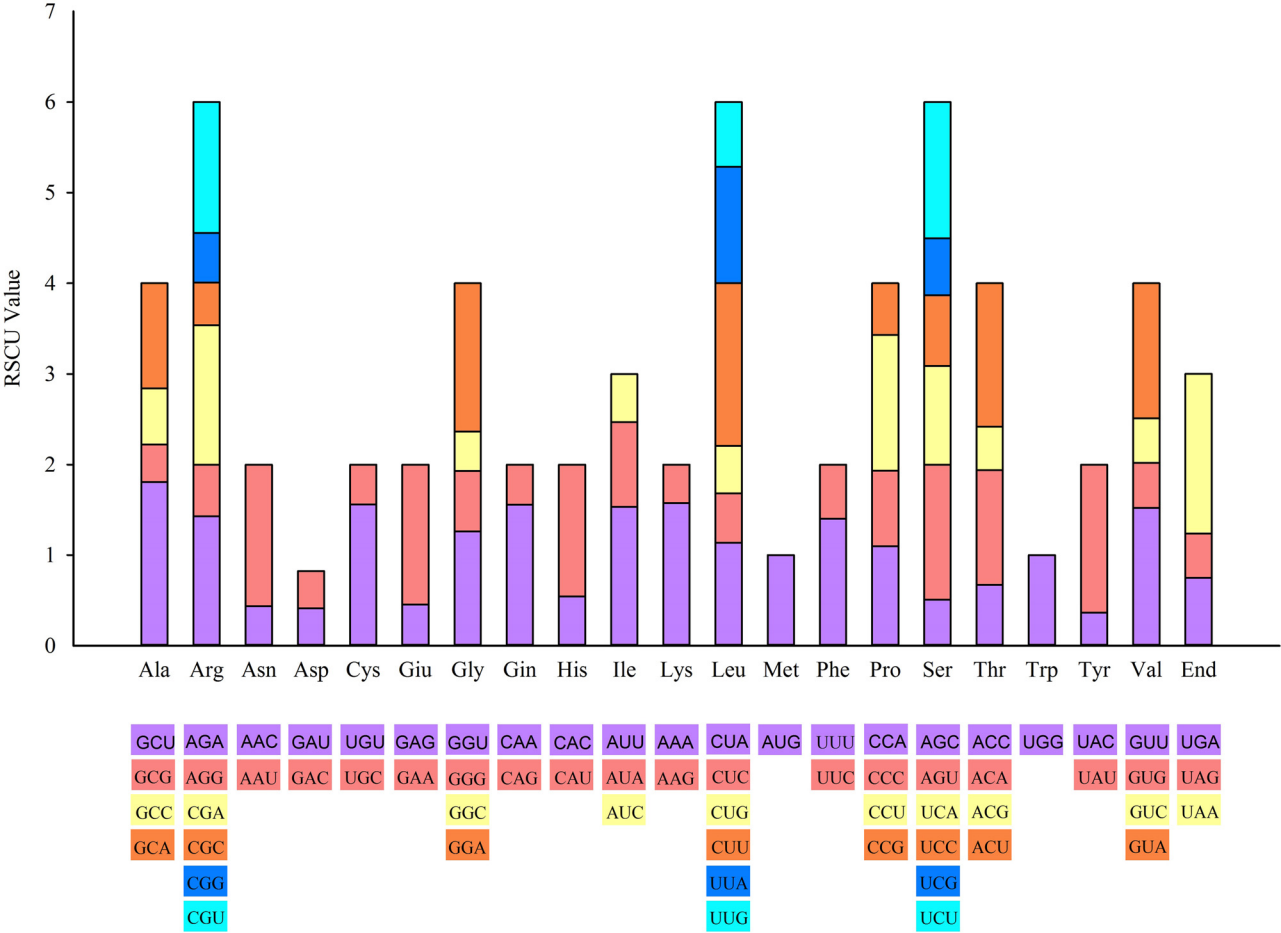
RSCU value of amino acid and stop codon in the chloroplast genome of *D. caryophyllus.* The color of the histogram corresponds to the color of the codon.

**Figure 8:**
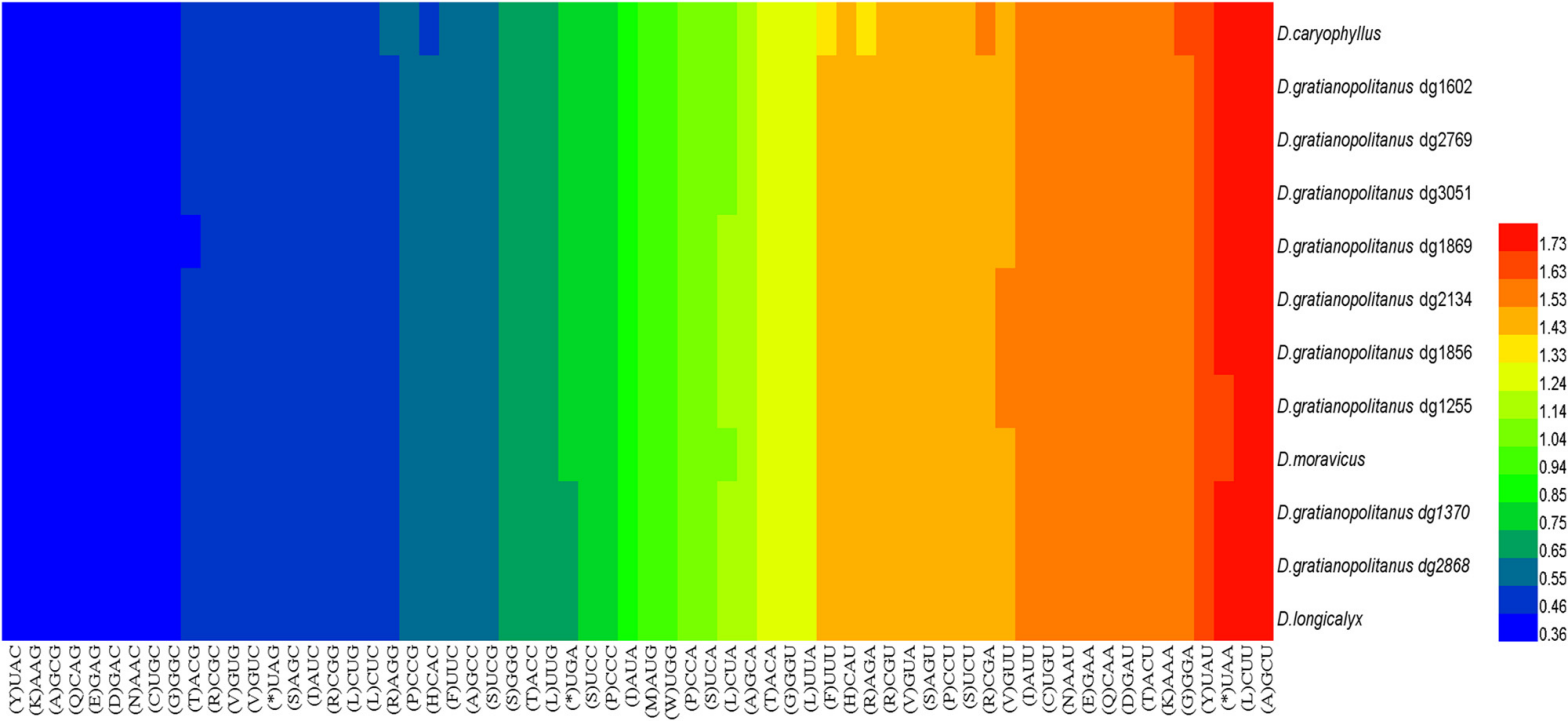
Heat map of codons usage of 12 species. Different colors represent different RSCU values, red indicates a higher RSCU value and blue indicates a lower RSCU value.

The codon usage bias of the 20 amino acids and stop codons in *D. caryophyllus* is depicted by the RSCU value (Figure 7). The RSCU value of each synonymous codon exhibited significant variation. Nearly all of the amino acids in protein-coding regions contained synonymous codons, except methionine and tryptophan. Among these, arginine and leucine displayed the highest codon usage preferences, with identical RSCU values, followed by serine. The heat map of all 12 species reveals variations in codon usage preferences between species and varieties (Figure 8). Notably, more than half of the codons exhibited RSCU values greater than 1. Additionally, we observed a preference for codons ending with A or U over those ending with C or G.

### 3.5 Evolutionary rate analyses

**Figure 9:**
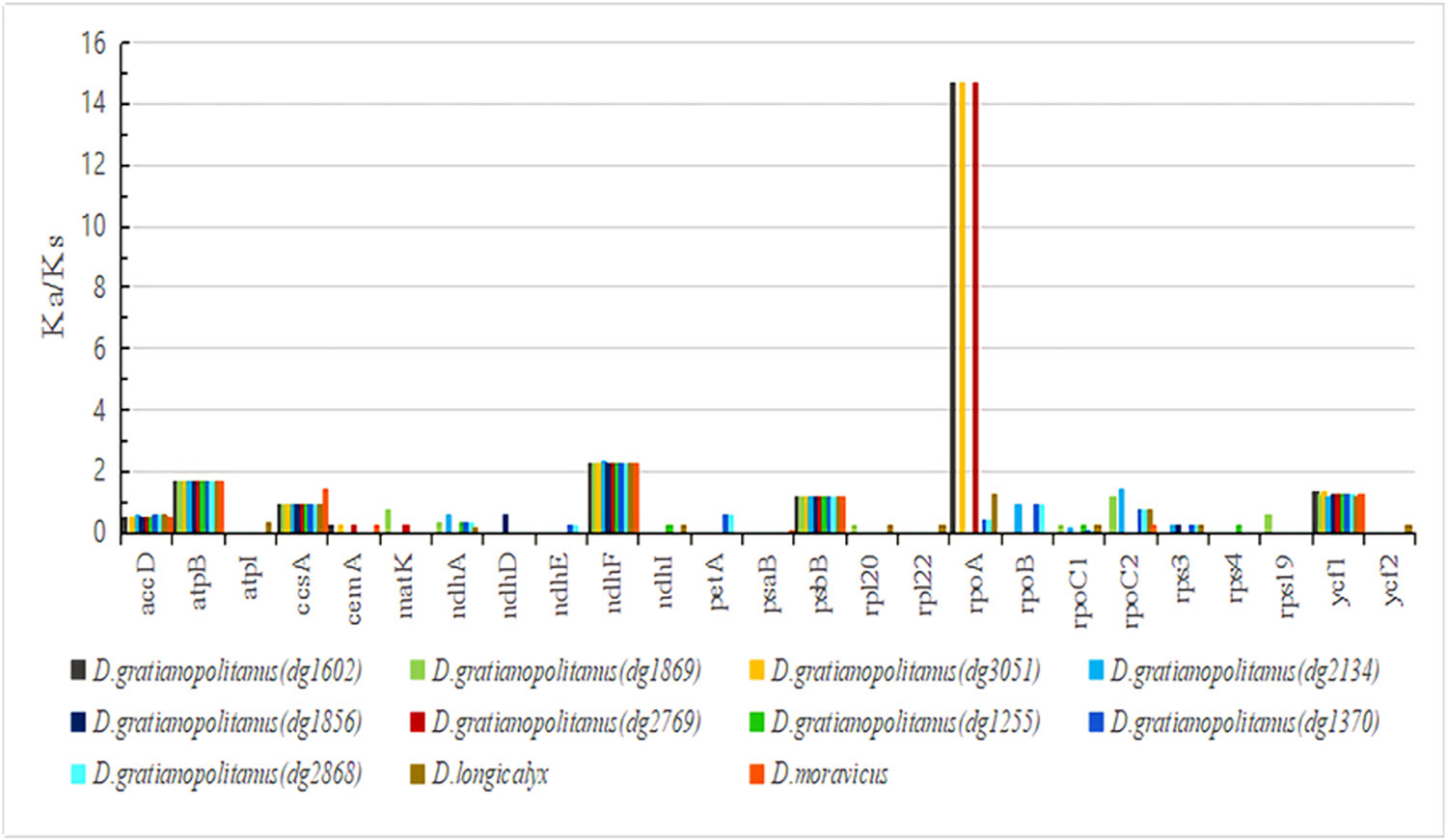
Ka/Ks comparison of protein genes with selective stress. The Ka/Ks values of other unlisted genes are zero.

In the evolutionary comparison of protein-coding genes with these 11 species (Figure 9), we observed that the Ka/Ks ratios among the nine *D. gratianopolitanus* varieties were similar. A significant proportion of protein-coding genes had Ka/Ks ratios less than 1, indicating that the non-synonymous sites were fewer than the synonymous sites, and these protein-coding genes underwent purify selection. Among all these species, only four genes (*atpB*, *ndhF*, *psbB*, and *ycf1*) had a value greater than 1, indicating positive selection. Comparatively, the *ccsA* of *D. moravicus* had a ratio greater than 1, whereas the ratios of the other species were slightly less than 1. A similar pattern was observed for *rpoC2* of *D. gratianopolitanus* (dg1869) and *D. gratianopolitanus* (dg2134). Additionally, none of the protein-coding genes exhibited evidence of neutral evolution. Worth noting is that the Ka/Ks ratios of *rpoA*, associated with RNA polymerases in *D. gratianopolitanus* (dg1602), *D. gratianopolitanus* (dg3051), and *D. gratianopolitanus* (dg2769) varieties were notably high, indicating strong positive selection.

### 3.6 Phylogenetic analysis

**Figure 10:**
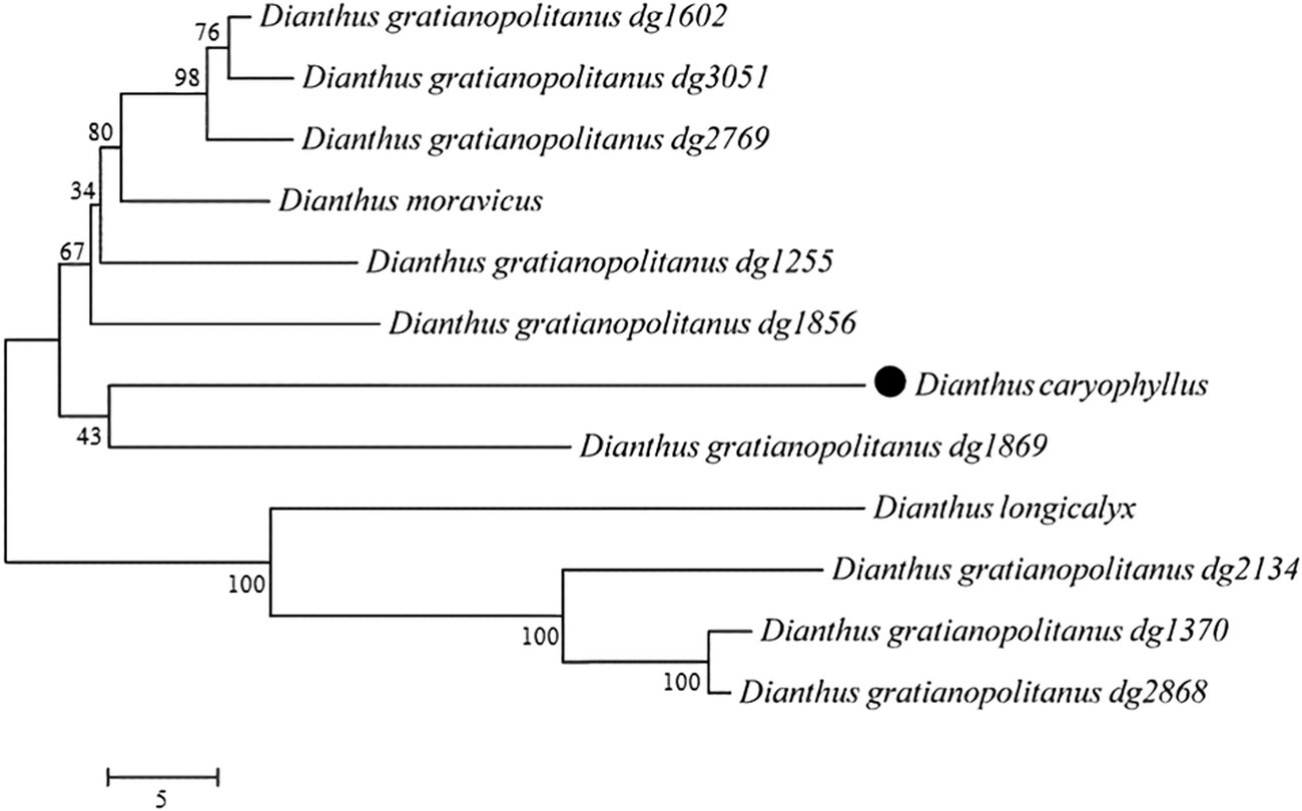
Phylogenetic analysis of the *D. caryophyllus* and 11 close relationship species by neighbor-joining method. The number indicates the NJ bootstrap value. The ruler indicates the genetic distance.

Phylogenetic analysis is a common method for studying species evolution and phylogenetic classification, forming the core of our understanding of biodiversity, evolution, and genomics. The phylogenetic tree divided these species into three major evolutionary branches (Figure 9). Specifically, *D. gratianopolitanus* (dg2134), *D. gratianopolitanus* (dg1370), *D. gratianopolitanus* (dg2868), and *D. longicalyx* formed one evolutionary branch, with a 100% bootstrap value. Meanwhile, *D. caryophyllus* and *D. gratianopolitanus* (dg1869) were divided into two main branches that diverged from another evolutionary branch. Similar to the trend of the RSCU cluster trend, although most plants within a species exhibited similarity, some plants showed closer evolutionary relationships between species (Figure 10).

## 4 Conclusion

In this study, we analyzed the structure, size, number, and type of genes and the codon usage and repeat sequences of CpDNA for 12 *Dianthus* species, including nine different varieties of *D. gratianopolitanus.* The size and GC content of CpDNA were similar among *Dianthus* species. Notably, *D. caryophyllus* had the smallest cpDNA, with significant contraction, but possessed the largest LSC region. *D. caryophyllus* was an exception, having 83 protein-coding genes because of the lack of the *rpl2* gene, whereas the other 11 species had all 84 protein-coding genes. Comparative analysis showed that the 12 CpDNAs of *Dianthus* species were highly conserved and that only a few sites of subtle variation existed. Gene analysis of the boundary regions showed that there were evident differences in the genes. The hypothesis is not supported by the data, as the differences within species were not less than the intra-species differences. More CpDNA data from *Dianthus* plants may be needed to explore molecular markers for distinguishing between different species.

## References

[1] Tanase K, Nishitani C, Hirakawa H, Isobe S, Tabata S, Ohmiya A, et al. Transcriptome analysis of carnation (Dianthus caryophyllus L.) based on next-generation sequencing technology. BMC Genomics. 2012;13(1):292. 10.1186/1471-2164-13-292 PMC3411436 22747974

[2] Chandra S, Rawat DS. Medicinal plants of the family Caryophyllaceae: a review of ethno-medicinal uses and pharmacological properties. Integr Med Res. 2015;4(3):123–31. 10.1016/j.imr.2015.06.004 PMC5481791 28664118

[3] Mutlu K, Sarikahya NB, Yasa I, Kirmizigul S. Dianthus erinaceus var. erinaceus: extraction, isolation, characterization and antimicrobial activity investigation of novel saponins. Phytochemistry Lett. 2016;16:219–24.

[4] Guo X-M, Yu Y-Y, Bai L, Gao R-F. Dianthus chinensis L.: the structural difference between vascular bundles in the placenta and ovary wall suggests their different origin. Front Plant Sci. 2017;8:1986. 10.3389/fpls.2017.01986 PMC5714885 29250086

[5] Fu X, Ning G, Gao L, Bao M. Genetic diversity of Dianthus accessions as assessed using two molecular marker systems (SRAPs and ISSRs) and morphological traits. Sci Horticulturae. 2008;117(3):263–70.

[6] Yagi M, Shirasawa K, Waki T, Kume T, Isobe S, Tanase K, et al. Construction of an SSR and RAD marker-based genetic linkage map for carnation (Dianthus caryophyllus L.). Plant Mol Biol Report. 2017;35(1):110–7.

[7] Wójcik M, Dresler S, Tukiendorf A. Physiological mechanisms of adaptation of Dianthus carthusianorum L. to growth on a Zn–Pb waste deposit – the case of chronic multi-metal and acute Zn stress. Plant Soil. 2015;390(1):237–50.

[8] Noman A, Aqeel M, Deng J, Khalid N, Sanaullah T, Shuilin H. Biotechnological advancements for improving floral attributes in ornamental plants. Front Plant Sci. 2017;8:530. 10.3389/fpls.2017.00530 PMC5397496 28473834

[9] Casanova E, Valdés AE, Zuker A, Fernández B, Vainstein A, Trillas MI, et al. rolC-transgenic carnation plants: adventitious organogenesis and levels of endogenous auxin and cytokinins. Plant Sci. 2004;167(3):551–60.

[10] Hu S, Ding Y, Zhu C. Sensitivity and responses of chloroplasts to heat stress in plants. Front Plant Sci. 2020;11:11. 10.3389/fpls.2020.00375 PMC7142257 32300353

[11] Zimorski V, Ku C, Martin WF, Gould SB. Endosymbiotic theory for organelle origins. Curr Open Microbiol. 2014;22:38–48. 10.1016/j.mib.2014.09.008 25306530

[12] Ravi V, Khurana JP, Tyagi AK, Khurana P. An update on chloroplast genomes. Plant Syst Evol. 2008;271(1):101–22.

[13] Li C, Zhao Y, Xu Z, Yang G, Peng J, Peng X. Initial characterization of the chloroplast genome of Vicia sepium, an important wild resource plant, and related inferences about its evolution. Front Genet. 2020;11:73. 10.3389/fgene.2020.00073 PMC7044246 32153639

[14] Powell W, Morgante M, McDevitt R, Vendramin GG, Rafalski JA. Polymorphic simple sequence repeat regions in chloroplast genomes: applications to the population genetics of pines. Proc Natl Acad Sci. 1995;92(17):7759–63. 10.1073/pnas.92.17.7759 PMC41225 7644491

[15] Stegemann S, Hartmann S, Ruf S, Bock R. High-frequency gene transfer from the chloroplast genome to the nucleus. Proc Natl Acad Sci U S A. 2003;100(15):8828–33. 10.1073/pnas.1430924100 PMC166398 12817081

[16] Martin W, Stoebe B, Goremykin V, Hansmann S, Hasegawa M, Kowallik KV. Gene transfer to the nucleus and the evolution of chloroplasts. Nature. 1998;393(6681):162–5. 10.1038/30234 11560168

[17] Peng J, Zhao Y, Dong M, Liu S, Hu Z, Zhong X, et al. Exploring the evolutionary characteristics between cultivated tea and its wild relatives using complete chloroplast genomes. BMC Ecol Evolution. 2021;21(1):71. 10.1186/s12862-021-01800-1 PMC8086295 33931026

[18] Park S, Jansen RK, Park S. Complete plastome sequence of Thalictrum coreanum (Ranunculaceae) and transfer of the rpl32 gene to the nucleus in the ancestor of the subfamily Thalictroideae. BMC Plant Biol. 2015;15(1):40. 10.1186/s12870-015-0432-6 PMC4329224 25652741

[19] Choi K-S, Park S. Complete plastid and mitochondrial genomes of Aeginetia indica reveal intracellular gene transfer (IGT), horizontal gene transfer (HGT), and cytoplasmic male sterility (CMS). Int J Mol Sci. 2021;22(11):22. 10.3390/ijms22116143 PMC8201098 34200260

[20] Li J, Li J, Ma Y, Kou L, Wei J, Wang W. The complete mitochondrial genome of okra (Abelmoschus esculentus): using nanopore long reads to investigate gene transfer from chloroplast genomes and rearrangements of mitochondrial DNA molecules. BMC Genomics. 2022;23(1):481. 10.1186/s12864-022-08706-2 PMC9245263 35768783

[21] Dobrogojski J, Adamiec M, Luciński R. The chloroplast genome: a review. Acta Physiol Plant. 2020;42(6):98.

[22] Sheng J, Yan M, Wang J, Zhao L, Zhou F, Hu Z, et al. The complete chloroplast genome sequences of five Miscanthus species, and comparative analyses with other grass plastomes. Ind Crop Products. 2021;162:113248.

[23] Wang X, Zhang R, Yun Q, Xu Y, Zhao G, Liu J, et al. Comprehensive analysis of complete mitochondrial genome of Sapindus mukorossi Gaertn.: an important industrial oil tree species in China. Ind Crop Products. 2021;174:114210.

[24] Chong X, Li Y, Yan M, Wang Y, Li M, Zhou Y, et al. Comparative chloroplast genome analysis of 10 Ilex species and the development of species-specific identification markers. Ind Crop Products. 2022;187:115408.

[25] Xu W, Lu R, Li J, Xia M, Chen G, Li P. Comparative plastome analyses and evolutionary relationships of all species and cultivars within the medicinal plant genus Atractylodes. Ind Crop Products. 2023;201:116974.

[26] Zhao J, Chen J, Xiong Y, He W, Xiong Y, Xu Y, et al. Organelle genomes of Indigofera amblyantha and Indigofera pseudotinctoria: comparative genome analysis, and intracellular gene transfer. Ind Crop Products. 2023;198:116674.

[27] Hu D, Zhang X, Xue P, Nie Y, Liu J, Li Y, et al. Exogenous melatonin ameliorates heat damages by regulating growth, photosynthetic efficiency and leaf ultrastructure of carnation. Plant Physiol Biochem. 2023;198:107698. 10.1016/j.plaphy.2023.107698 37060867

[28] Chen S, Xu Z, Zhao Y, Zhong X, Li C, Yang G. Structural characteristic and phylogenetic analysis of the complete chloroplast genome of Dianthus caryophyllus. Mitochondrial DNA Part B-Resources. 2018;3(2):1131–2. 10.1080/23802359.2018.1521313 PMC7799632 33474443

[29] Putz CM, Schmid C, Reisch C. Living in isolation – population structure, reproduction, and genetic variation of the endangered plant species Dianthus gratianopolitanus (Cheddar pink). Ecol Evolution. 2015;5(17):3610–21. 10.1002/ece3.1611 PMC4567865 26380690

[30] Greiner S, Lehwark P, Bock R. Organellar genome DRAW (OGDRAW) version 1.3.1: expanded toolkit for the graphical visualization of organellar genomes. Nucleic Acids Res. 2019;47(W1):W59–64. 10.1093/nar/gkz238 PMC6602502 30949694

[31] Hall T. BioEdit: a user-friendly biological sequence alignment editor and analysis program for windows 95/98/NT. Nucleic Acids Symp Ser. 1999;41:95–8.

[32] Frazer KA, Pachter L, Poliakov A, Rubin EM, Dubchak I. VISTA: computational tools for comparative genomics. Nucleic Acids Res. 2004;32(suppl_2):W273–W9. 10.1093/nar/gkh458 PMC441596 15215394

[33] Alikhan N-F, Petty NK, Ben Zakour NL, Beatson SA. BLAST ring image generator (BRIG): simple prokaryote genome comparisons. BMC Genomics. 2011;12(1):402. 10.1186/1471-2164-12-402 PMC3163573 21824423

[34] Zhang W, Zhao Y, Yang G, Peng J, Chen S, Xu Z. Determination of the evolutionary pressure on Camellia oleifera on Hainan Island using the complete chloroplast genome sequence. Peerj. 2019;7:e8115. 10.7717/peerj.7210 PMC6599451 31289703

[35] Kurtz S, Choudhuri JV, Ohlebusch E, Schleiermacher C, Stoye J, Giegerich R. REPuter: the manifold applications of repeat analysis on a genomic scale. Nucleic Acids Res. 2001;29(22):4633–42. 10.1093/nar/29.22.4633 PMC92531 11713313

[36] Beier S, Thiel T, Münch T, Scholz U, Mascher M. MISA-web: a web server for microsatellite prediction. Bioinformatics. 2017;33(16):2583–5. 10.1093/bioinformatics/btx198 PMC5870701 28398459

[37] Xia X, Xie Z. DAMBE: Software package for data analysis in molecular biology and evolution. J Heredity. 2001;92(4):371–3. 10.1093/jhered/92.4.371 11535656

[38] Deng W, Wang Y, Liu Z, Cheng H, Xue Y. HemI: a Toolkit for illustrating heatmaps. PLoS One. 2014;9(11):e111988. 10.1371/journal.pone.0111988 PMC4221433 25372567

[39] Librado P, Rozas J. DnaSP v5: a software for comprehensive analysis of DNA polymorphism data. Bioinformatics. 2009;25(11):1451–2. 10.1093/bioinformatics/btp187 19346325

[40] Kumar S, Stecher G, Tamura K. MEGA7: molecular evolutionary genetics analysis version 7.0 for bigger datasets. Mol Biol Evolution. 2016;33(7):1870–4. 10.1093/molbev/msw054 PMC8210823 27004904

